# Design of polybenzimidazolium membranes for use in vanadium redox flow batteries†

**DOI:** 10.1039/d3ta07212f

**Published:** 2024-02-08

**Authors:** J. C. Duburg, B. Chen, S. Holdcroft, T. J. Schmidt, L. Gubler

**Affiliations:** a Electrochemistry Laboratory, Paul Scherrer Institut CH-5232 Villigen PSI Switzerland lorenz.gubler@psi.ch; b Simon Fraser University, Department of Chemistry V5A 1S6 Burnaby Canada; c Institute of Molecular Physical Science, ETH Zurich CH-8093 Zurich Switzerland

## Abstract

In recent years, polybenzimidazole (PBI) membranes have been proposed for vanadium redox flow batteries (VRFBs) as an alternative to perfluoroalkylsulfonic acid membranes such as Nafion™. Despite their excellent capacity retention, PBI membranes tend to suffer from a low ionic conductivity. The formation of a polybenzimidazolium through an *N*-alkylation of the benzimidazole core is shown to improve the ionic conductivity of the membrane, with this class of materials having found uses in alkaline fuel cell and water electrolysis systems. However, much less is known about their incorporation into a VRFB. This article describes the use of hexamethyl-*p*-terphenyl polybenzimidazolium (HMT-PMBI) membranes for a vanadium redox flow battery, with the membrane characteristics in acidic media being related to their performance in a single-cell VRFB setup. A change of the degree of methylation from 56 to 65, 75, and 89% leads to an increase in ionic conductivity, correlated with an increased fraction of free water in the ionomer. The corresponding increase in cell performance is, however, accompanied by a drop in capacity retention. The membrane with a degree of methylation of 65% shows balanced properties, with a 5% higher efficiency and a two times improved capacity retention compared to Nafion™ NR212 over 200 charge–discharge cycles at 200 mA cm^−2^.

## Abstract 2

Hexamethyl-*p*-terphenyl polybenzimidazolium (HMT-PMBI) for a vanadium redox flow battery (VRFB). A higher degree of methylation increases the ionic conductivity of the polymer electrolyte but reduces its capacity retention in a VRFB.

## Graphical abstract

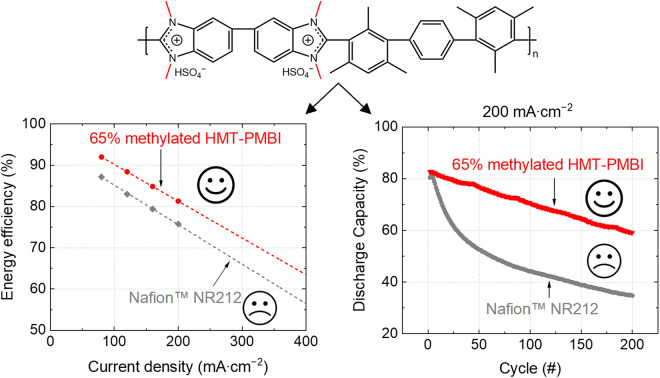

## 1 Introduction

**Fig. 1:**
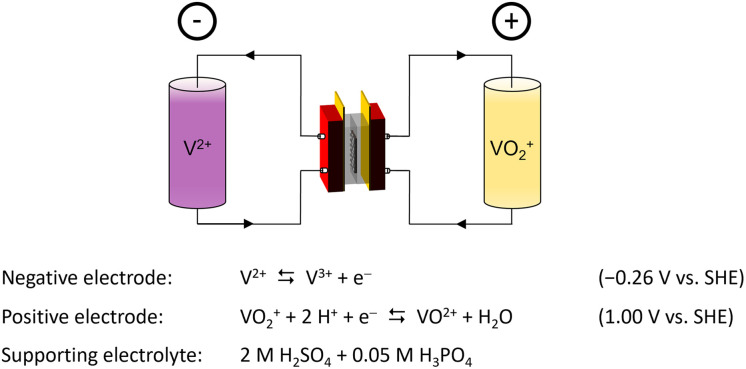
Schematic depiction of a vanadium redox flow battery in its charged state.

Nowadays, the demand for highly efficient and low-cost energy storage technologies to combat climate change and to assist the inherently intermittent energy technologies of solar and wind power is growing rapidly. In doing so, the flexibility to independently scale energy capacity and power output is highly sought after to enable their large-scale implementation into our energy infrastructure. One of the energy storage technologies able to do so are redox flow batteries (RFBs), of which there are several types.^1,2^ At the moment, the most advanced RFB is based on a vanadium redox couple at both electrodes, Fig. 1. As a result of the identical electroactive material, the vanadium redox flow battery (VRFB) reduces the risk of cross contamination during operation,^3^ which enables electrolyte recycling and avoids the irreversible loss of capacity.^4^ Here, a liquid electrolyte, containing the electroactive vanadium ions, is circulated between an external electrolyte storage tank and the internal compartments of an electrochemical cell, contributing to the separation of energy capacity and power output, respectively.^5^ Conventionally, VRFBs are equipped with membranes made from perfluorinated sulfonic acid polymers (PFSAs), such as Nafion™.^6,7^ Although these materials demonstrate excellent chemical stability and ionic conductivity, their incorporation in large-scale VRFB systems is hindered by their poor capacity retention and high cost.^8–11^ Furthermore, their fluorine containing backbone is of great concern due to the health risks associated to the fabrication and degradation of the material.^12–14^ As an alternative to PFSAs, low-cost polymers based on polybenzimidazole (PBI), such as *meta*-polybenzimidazole (*m*-PBI), exhibiting excellent vanadium barrier properties have been put forward.^15–17^ In a VRFB, the basic benzimidazole moiety of the PBI polymer reacts with the acidic electrolyte, gaining the properties of an anion exchange membrane through the formation of a fixed positive charge on the polymer backbone.^18^ Although thin *m*-PBI membranes were proven to be excellent candidates for use in VRFB systems, their limited conductivity hindered their incorporation as thicker stand-alone membranes.^16,17^ To tackle this, several strategies were proposed to further improve the performance of benzimidazole membranes in VRFBs. These strategies can be divided into three main categories: (i) the reduction of membrane thickness, resulting in the need of porous materials providing mechanical support to the PBI skin layer,^19–22^ (ii) the increase of porosity and/or free volume of the polybenzimidazole membrane,^23–26^ thereby increasing the swelling and uptake characteristics of the membrane, and lastly (iii) the chemical modification of the benzimidazole core, providing an improved ionic conductivity and chain separation of the polymer.^27–31^ In the latter, the typical approach is to introduce ionically charged pendant chains to the polymer backbone that form the basis of charged channels within the membrane. Doing so, Yan *et al.* introduced a sulfonic acid-terminated sidechain onto a diphenylether–PBI backbone, thereby creating proton conducting moieties and enabling the formation of a 55 μm thick PBI based membrane that outperforms Nafion™ NR212 in both energy efficiency and capacity retention.^29^ Pang *et al.* adopted this work and introduced a quaternary ammonium pendant chain alongside a sulfate ester, creating an amphoteric membranes with an improved capacity retention.^31^ In a different approach, the charge on the polymer can also be created on the PBI backbone itself. Here, the formation of a polybenzimidazolium through an *N*-alkylation of the benzimidazole core on both nitrogen positions is shown to greatly enhance the conductivity of the membrane.^32,33^ In recent years, polybenzimidazolium membranes have been shown to be a very promising material for membranes in alkaline water electrolysis and alkaline fuel cell systems due to their excellent ionic conductivity and chemical stability.^33–35^ One of these materials is hexamethyl-*p*-terphenyl polybenzimidazolium (HMT-PMBI), which was first synthesized and reported by Wright *et al.* in 2014.^32,33^ Here, a 2,2′′,4,4′′,6,6′′-hexamethyl-*p*-terphenylene (HMT) was added alongside the polybenzimidazolium core to increase the hydrophobicity of the polymer, while maintaining the C2-protection of the cation. In doing so, a polymer with a good alkaline stability was obtained that could easily be produced at a large scale, as shown by Wright *et al.* in 2016,^33^ thereby forming the basis of the trademarked Aemion™ produced by Ionomr Innovations Inc.^36^ Despite the strong performances seen for both Aemion™ and the pristine HMT-PMBI in alkaline media, little is known about their performance and the role of the degree of methylation in acidic media such as a VRFB. Thus far, the studies done on HMT-PMBI and Aemion™ for VRFBs focused on a singular type of HMT-PMBI^37^ or commercial variants of Aemion™ with an unknown chemical structure.^36,38^ With this in mind, this article describes an in-depth analysis of the performance and characteristics of HMT-PMBI with a varying degree of methylation in the acidic conditions of a VRFB. Here, the degree of methylation of the membrane is related to characteristics such as the water and acid uptake, ionic conductivity and permeability, providing insight in the observed performance of the membrane in a VRFB cell. Furthermore, the acquired knowledge can form the basis of a deeper understanding between the molecular structure of the membrane and the expected performance, thereby enabling the future optimization of this class of materials for operation under VRFB conditions.

## 2 Experimental

### 2.1. HMT-PMBI

**Fig. 2:**
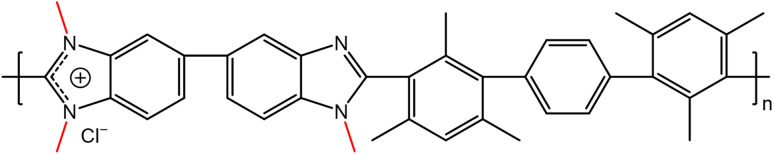
Chemical structure of HMT-PMBI with a degree of methylation of 75% (75dm). The methyl pendant groups are highlighted in red as visual aid.

Hexamethyl-*p*-terphenyl polybenzimidazolium polymers with different degrees of methylation of 56% (56dm), 65% (65dm), 75% (75dm) and 89% (89dm) were prepared by the Holdcroft group,^32,33^ with the chemical structure of HMT-PMBI with a degree of methylation of 75% shown in Fig. 2. The degree of methylation of each polymer was verified using ^1^H nuclear magnetic resonance (NMR) spectroscopy as described by Wright *et al.*^33^ with these results shown in the ESI.†

### 2.2. Membrane preparation

HMT-PMBI membranes of 56dm, 65dm and 75dm were prepared by solution casting and the subsequent evaporation of the casting solvent, with the 89dm membranes already present in the Holdcroft group. Prior to the preparation of the casting solution, the polymeric powders were dissolved in dichloromethane (56dm and 65dm) or methanol (75dm) and filtered through a paper filter. The polymer was recovered by partially covering the filtrate, allowing for a slow evaporation of the solvent, with the dry polymer being obtained after a second drying step at 80 °C *in vacuo* overnight. The casting solution was prepared by dissolving the polymer at 80 °C in *N*-methyl-2-pyrrolidone (56dm and 65dm) or dimethylsulfoxide (75dm). Subsequently, the membranes were prepared through casting of the polymeric solution on a glass plate using an adjustable applicator, followed by the evaporation of the casting solvent at 80 °C overnight in a Shel Lab convection oven. For the detailed membrane casting conditions, please see Table S1.† After drying, the membranes, including the 89dm film, were allowed to cool down to room temperature and placed in 3 M sodium chloride for 3 days, with the solution being refreshed every 24 h. The final membranes were obtained after washing with Milli-Q water to remove excess sodium chloride and drying at 80 °C *in vacuo* overnight.

### 2.3. XRF analysis

The influence of the acidic electrolyte on the molecular structure of HMT-PMBI with respect to the counter-ion of the polymer was analyzed with X-ray fluorescence (XRF). In this process, an 89dm HMT-PMBI sample was placed in 2 M H_2_SO_4_ for two days, with the solution being refreshed after the first day. Subsequently, the film was washed multiple times over a 24 h period with Milli-Q water until a stable pH was reached and dried at 80 °C *in vacuo* overnight. The treated film was then placed on top of an XRF sample holder and its chemical composition was analyzed for its carbon, chlorine, iodine and sulfur content. Subsequently the CH_2_ content was inferred from the measured carbon content. The detection limits of the XRF analysis can be seen in Table S2.†

### 2.4. Dimensional swelling and electrolyte uptake

The membrane swelling and electrolyte uptake in 2 M H_2_SO_4_ of the various HMT-PMBI membranes was measured through the analysis of four circular samples (*Ø* 3.0 cm). The samples were dried at 80 °C *in vacuo* overnight. Subsequently, the dry weight of the membrane, *m*_dry_, was measured in a glass vial with a known weight to avoid the imbibition of moisture from the air before measuring the dry thickness and diameter, respectively *t*_dry_ and *d*_dry_, of the material. After immersion in 2 M H_2_SO_4_ for a minimum of 16 h, the swollen thickness and diameter, respectively *t*_wet_ and *d*_wet_, of the membrane were determined. Subsequently, the samples were placed back into a fresh 2 M H_2_SO_4_ solution for a minimum of 4 h, before measuring their swollen weight in a glass vial with a known mass to avoid the drying of the film. The swollen membranes were dried overnight at 80 °C *in vacuo* to remove the water content inside the film. Afterwards, the dried weight of the membrane, *m*_dried_, corresponding to its pure H_2_SO_4_ swollen state, was measured by placing the dried membrane onto the balance, with the weight being recorded after 3 seconds to minimize the uptake of moisture from the air. The dimensional swelling and the total mass, H_2_O and H_2_SO_4_ uptake in 2 M H_2_SO_4_ were calculated according to eqn (1)–(5). In addition, the bound HSO_4_^−^ and the ion-exchange capacity (IEC) of HMT-PMBI were calculated from their corresponding ^1^H NMR spectrum according to eqn (6) and (7), with dm as degree of methylation and *M*_HMT-PMBI_ as the molecular weight of HMT-PMBI.1
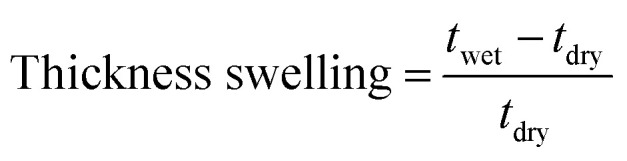
2
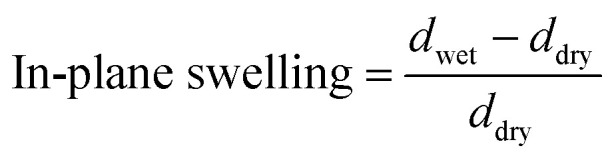
3
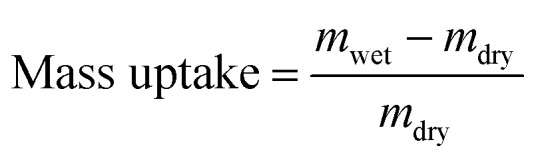
4
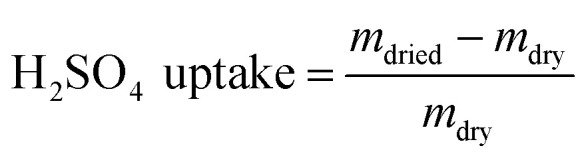

5 H_2_O uptake = mass uptake − H_2_SO_4_ uptake

6
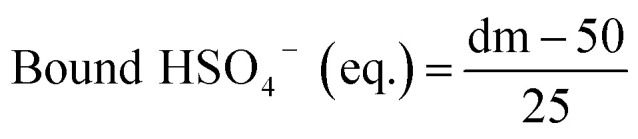
7
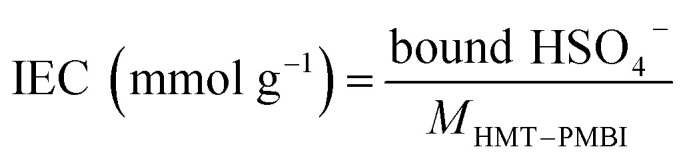


### 2.5. Through-plane ionic conductivity

The through-plane ionic conductivity of the HMT-PMBI variants was determined through the analysis of five samples (1.0 × 1.0 cm). The samples were placed in 2 M H_2_SO_4_ or 1.6 M vanadium electrolyte (average oxidation state 3.5, GFE, Germany) overnight to mimic the VRFB operating conditions. Subsequently, the resistance of the membrane was measured in a through-plane setup, equipped with two platinum electrodes (0.5 × 0.5 cm) and a Solartron SI 1260 impedance analyzer. Electrochemical impedance spectroscopy (EIS) spectra of the singular samples were recorded between 100 Hz and 1 MHz and normalized by subtracting the inductance and resistance of the blank cell. The high-frequency intercept (HFR) was obtained from the normalized spectra where the imaginary axis equals 0. If the intercept of the semi-circle was not reached, a linear fit of the last data points was used to obtain the HFR. The ionic conductivity in the swollen state was calculated according to eqn (8), with *t*_wet_ as the swollen thickness, measured after recording the EIS spectrum.8
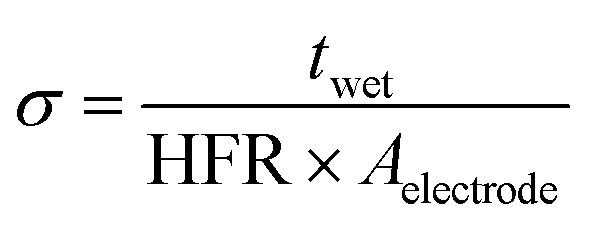


### 2.6. VO^2+^ diffusion

The VO^2+^ diffusion coefficient of the HMT-PMBI membranes was determined in a custom-made glass H-cell with an active area of 10.2 cm^2^ (Fig. S6†). All samples were conditioned in 2 M H_2_SO_4_ for 24 h prior to analysis. The membranes were placed in between a VO^2+^ donating compartment (1.6 M VOSO_4_ in 2 M H_2_SO_4_, 80 mL) and a VO^2+^ receiving compartment (1.6 M MgSO_4_ in 2 M H_2_SO_4_, 80 mL), with both compartments continuously stirred to avoid any concentration imbalances. Aliquots of the receiving compartment were analyzed daily over a period of 7–10 days by UV-Vis spectroscopy (Agilent Cary 4000 spectrophotometer). The absorbance of the receiving compartment was recorded between a wavelength of 500 nm and 800 nm by filling a quartz cuvette (10 mm, Hellma Analytics) with 3 mL aliquots. From this, the VO^2+^ concentration was calculated using a VO^2+^ calibration curve (Fig. S5†) at a wavelength of 765 nm. Subsequently, the receiving compartment was replenished with the taken aliquots to avoid volume changes. If the absorbance of VO^2+^ in the receiving compartment exceeded 1.1, 0.5 mL aliquots were taken instead and diluted to 3 mL with 1.6 M MgSO_4_ in 2 M H_2_SO_4_. In this case, the aliquots were not placed back into the receiving compartment and instead 0.5 mL was removed from the VO^2+^ donating compartment to ensure an equal volume on both sides. The VO^2+^ diffusion coefficient (*D*_VOSO4_) was calculated according to eqn (9), with *V*_r_ the volume of the receiving compartment (80 mL), *A* the membrane surface area (10.2 cm^2^), *t* the swollen membrane thickness in cm, *C*_d_ the VO^2+^ concentration in the donating compartment (1.6 M) and *C*_r_ the VO^2+^ concentration in the receiving compartment at a given point in time.9
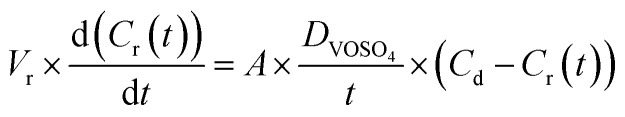


The reported values for the VO^2+^ diffusion coefficient were obtained from an average over 4 individual measurements, with the given error being the standard deviation between these tests.

### 2.7. Mechanical strength

The mechanical properties of the HMT-PMBI derivatives were obtained through an ASTM D638-5 protocol using a single column Instron 3344 test system. HMT-PMBI membranes were equilibrated overnight in a 1.6 M vanadium electrolyte (oxidation state 3.5, GFE, Germany) after which the samples to be analyzed were punched out using an ASTM D638-5 dumbbell punching tool. Prior to testing, the samples were stored in the 1.6 M vanadium electrolyte to avoid the drying out of the films. The tensile test was carried out at ambient conditions, with a minimum of three samples analyzed for each measurement to ensure reproducibility and the given error being standard deviation over these three measurements.

### 2.8. Chemical stability

The oxidative stability of the HMT-PMBI variants was assessed using a 1.6 M VO_2_^+^ in 2 M H_2_SO_4_ electrolyte. The electrolyte was obtained through the oxidization of the commercial 1.6 M vanadium electrolyte (average oxidation state 3.5, GFE, Germany), measured as 1.73 M vanadium by ICP-OES, in a single-cell VRFB setup equipped with FAP-330, a commercial anion exchange membrane (Fumatech, Germany). To ensure a complete oxidation of vanadium to VO_2_^+^ on the positive electrode, the positive electrolyte was made limiting through the addition of an excess of electrolyte on the negative side. The system was galvanostatically charged to 1.7 V by a stepwise decrease of the current density from 200 mA cm^−2^ to 40 mA cm^−2^. Subsequently, a constant voltage hold at 1.7 V allowed for the complete oxidation of VO^2+^ to VO_2_^+^, with the electrolyte being collected upon reaching a current density below 4 mA cm^−2^.

For each HMT-PMBI membrane, three samples (2.0 × 4.0 cm) were punched out and dried *in vacuo* at 70 °C overnight, after which their dry weight was measured. Subsequently, the chloride counter-ion in the membrane was exchanged by placing the samples in 2 M H_2_SO_4_ for 2 days, with the solution being refreshed after 24 h. The oxidative stability test was started by immersing the exchanged membranes in 5 mL of the VO_2_^+^ electrolyte, with the vials being purged with N_2_ before sealing. The VO^2+^ formation in the samples was determined after 25, 55 and 90 days at room temperature by diluting 0.1 mL of the electrolyte with 2.9 mL of 2 M H_2_SO_4_. Then, the VO^2+^ concentration was determined by UV-Vis spectroscopy as described in Section 2.6, with two VO_2_^+^ electrolyte reference solutions, prepared at the same time and stored under identical conditions acting as the VO^2+^ baseline. Post test, the membrane samples were washed in 2 M H_2_SO_4_ for two days, with the solution being refreshed after the first day, to remove residual vanadium from inside the film. Subsequently, the samples were placed in 3 M NaCl for three days, with the solution being refreshed each day, to remove residual acid and re-exchange the membrane to its chloride form. After three days, the films were washed in deionized water for one day and dried *in vacuo* at 80 °C overnight. The dried films were analyzed by Fourier-transform infrared spectroscopy (FTIR, Vertex V70 spectrometer, Bruker, USA) between a wavenumber of 400 cm^−1^ and 4000 cm^−1^ and ^1^H NMR spectroscopy in dimethylsulfoxide-d_6_.

### 2.9. VRFB cell cycling

The cycling performance of HMT-PMBI membranes was analyzed in an electrochemical test station (Scribner Model 857 test stand, Scribner Associates, USA) equipped with a laboratory electrochemical cell (25 cm^2^ active area, Scribner). Nafion™ NR212 (51 μm dry thickness, Ion Power, USA) was used as the commercial reference. The electrochemical test station was equipped with two in-house designed graduated cylindrical glass vessels and a multichannel peristaltic pump (Masterflex L/S^®^, GZ-07522-20, Cole-Parmer GmbH, Germany) using plasticizer-free chemical resistant tubing (Versilon™ 2001, GZ-06475-16, Cole-Parmer GmbH, Germany). The electrochemical cell assembly consisted of (i) two triple-serpentine cured furan resin graphite flow fields (Fuel Cell Technologies, Inc., USA), (ii) two gold-plated copper current collectors (Scribner Associates, USA), (iii) two pre-treated carbon felt electrodes (25 cm^2^ active area, AAF304ZS, Toyobo, Japan), used as received, (iv) two in-house designed polyvinylidene fluoride gasket frames (2.5 mm thickness) equipped with two ice cube gaskets (0.8 mm, 35 FC-PO100, Freudenberg, Germany). The electrochemical cell was closed with eight bolts and tightened to 4 N m (carbon felt compression of 42%). The graduated cylindrical tanks were filled with 40 mL of 1.6 M vanadium electrolyte, measured as 1.73 M by ICP-OES, in 2 M H_2_SO_4_ and 0.05 M H_3_PO_4_ (oxidation state 3.5, GFE, Germany) on each side and continuously flushed with argon (66 mL min^−1^). An experimentally measured flow rate of 60 mL min^−1^ was used to circulate the electrolyte during cycling.

Prior to cell assembly, the HMT-PMBI membranes were pre-swollen in 2 M H_2_SO_4_ overnight, with Nafion™ NR212 being equilibrated in deionized water to enhance its swelling. The swollen HMT-PMBI membranes were subjected to a galvanostatic cycling test at varying current densities. Herein, the pre-swollen membranes were conditioned in the vanadium electrolyte and the electrochemical cell for 4 h at OCV, after which the high-frequency intercept was measured with electrochemical impedance spectroscopy between 10 kHz and 0.1 Hz at zero DC and a perturbation amplitude of 10 mV. Following the conditioning phase, the electrolyte was galvanostatically charged at 40 mA cm^−2^ until the upper cut-off limit of 1.65 V was reached, with a polarization curve measured at a state of charge of 20, 30, 50, 70 and 90%. The polarization curves were recorded by consecutively charging and discharging the electrolyte at 20, 40, 60, 80, 120, 150 and 200 mA cm^−2^ for 20 s with 0.80 V and 1.80 V as the lower and upper potential limits. Upon fully charging the system, the cycling performance was analyzed through sequentially cycling the system at a constant current density of 80, 120, 160, 200 and 120 mA cm^−2^ between 0.80 V and 1.65 V for respectively, 10, 25, 10, 10 and 5 cycles. The 1^st^ cycle at each current density was excluded from the analysis due to the prior change in current density.

The polymers that performed well in the cell cycling test at varying current densities were further analyzed in an extended cycling test at a current density of 200 mA cm^−2^. As before, the pre-swollen membranes were conditioned in the vanadium electrolyte and electrochemical cell for 4 h at OCV before their high-frequency intercept was measured through electrochemical impedance spectroscopy between 10 kHz and 0.1 Hz at zero DC and a perturbation amplitude of 10 mV. After conditioning, the system was charged at 200 mA cm^−2^ until the upper cut-off limit of 1.65 V was reached. Subsequently, the VRFB was galvanostatically cycled at 200 mA cm^−2^ between 0.80 V and 1.65 V as the respective lower and upper potential limits for 200 cycles. The error in the measured coulombic efficiency was minimized by forming a moving average of the measured cycle with the two cycles before and after, with the 1^st^ cycle of each test being omitted. As a result of a potentiostat malfunction during the extended cycling test of 65dm, two cycles were omitted with an additional two cycles recorded at the end of the test. The cycling efficiencies and discharge capacity are calculated according to eqn (10)–(13), with *Q*_dis_ and *Q*_ch_ the capacity during discharging and charging, *Ū*_dis_ and *Ū*_ch_ the average potential during discharging and charging, *n* the number of moles of the electroactive vanadium species and *F* the Faraday constant (96 485 C mol^−1^). The electrolyte crossover was calculated according to eqn (14), where crossover_neg_ is the total electrolyte crossover towards the negative electrode, with *V*_end_^−^, *V*_end_^+^, *V*_start_^−^ and *V*_start_^+^ being the electrolyte volume in the negative and positive compartment at the end and start of the test. Subsequently, the fraction of electrolyte crossover was calculated according to eqn (15), with *V*_start_ as the electrolyte volume at the start of the test on both sides (40 mL).10
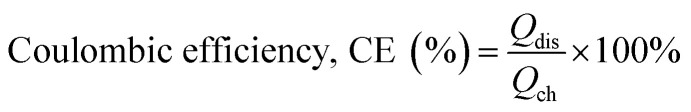
11
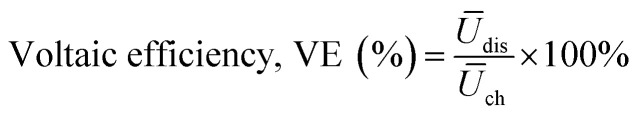

12 Energy efficiency, EE (%) = CE × VE

13
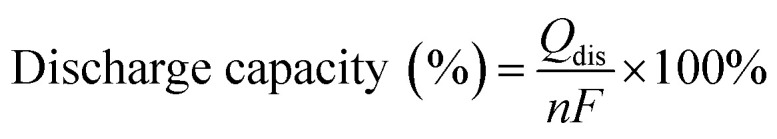
14
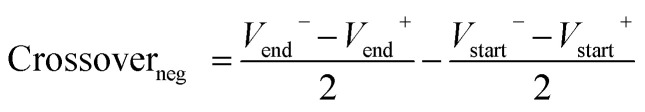
15
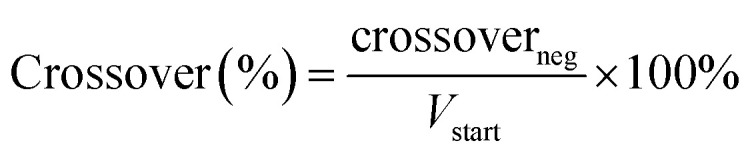


## 3 Results and discussion

In alkaline anion exchange membrane fuel cell (AEMFC) and water electrolysis (AEMWE) devices, HMT-PMBI membranes are used as an OH^−^ conducting ionomer. In AEMWE, often a KOH solution of low concentration (*e.g.* 1 M) is used as a supporting electrolyte for improved performance and durability.^39^ In the case of a VRFB system, the membrane is subjected to an acidic electrolyte comprising of the redox-active vanadium species in addition to the sulfuric acid supporting electrolyte. As the pH of the supporting electrolyte is lower than the p*K*_a_ of the partially deprotonated HSO_4_^−^ form (2–3 M H_2_SO_4_, pH < 0), the bulk of the supporting electrolyte will consist of HSO_4_^−^ and H^+^ species. Furthermore, since the pristine HMT-PMBI membrane contains Cl^−^ or I^−^ as counter-ion, an ion exchange to the HSO_4_^−^ form during pretreatment in the acidic electrolyte can occur. To confirm the ion exchange process, an 89dm membrane sample was subjected to a 2 M H_2_SO_4_ solution for two days, with the solution being refreshed after 24 h. Subsequently, the membrane was washed with DI water to remove the excess electrolyte and dried before analyzing the elemental composition of the film with XRF, Table 1.

**Table 1:** XRF analysis of pristine and treated 89dm

Component	Pristine 89dm (wt%)	Treated 89dm (wt%)
S	0.2	1.4
Cl	1.3	<0.1
I	0.1	<0.1
CH_2_	98.3	98.5

As expected, the non-treated membrane showed the presence of the halogenic Cl^−^ and I^−^ counter-ions. Upon treating the membrane in 2 M H_2_SO_4_, these ions were exchanged with a similar mass fraction of sulfur, thereby confirming that the membrane indeed undergoes a counter-ion exchange from its pristine Cl^−^ and I^−^ state to a HSO_4_^−^ form in the acidic medium of a VRFB. With this in mind, all membranes were conditioned for a minimum of 24 h in 2 M H_2_SO_4_ to introduce the appropriate counter-ion representative of the membrane during operation.

**Fig. 3:**
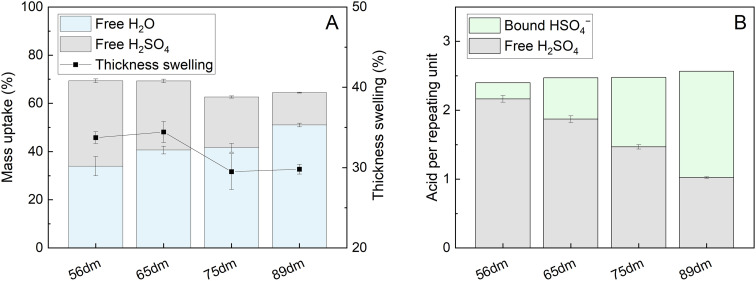
Water uptake, acid uptake and thickness swelling of HMT-PMBI membranes with various degrees of methylation (dm) upon immersion in 2 M H_2_SO_4_ (A) and the corresponding acid content per repeating unit (B).

With the known molecular structure of the polymer in acidic medium, the polymer electrolyte itself can be characterized. Here, the membrane was subjected to 2 M H_2_SO_4_ to analyze its affinity to the supporting electrolyte in terms of thickness swelling, water uptake and acid uptake (Fig. 3). These parameters were determined as described in Section 2.4.

**Fig. 4:**
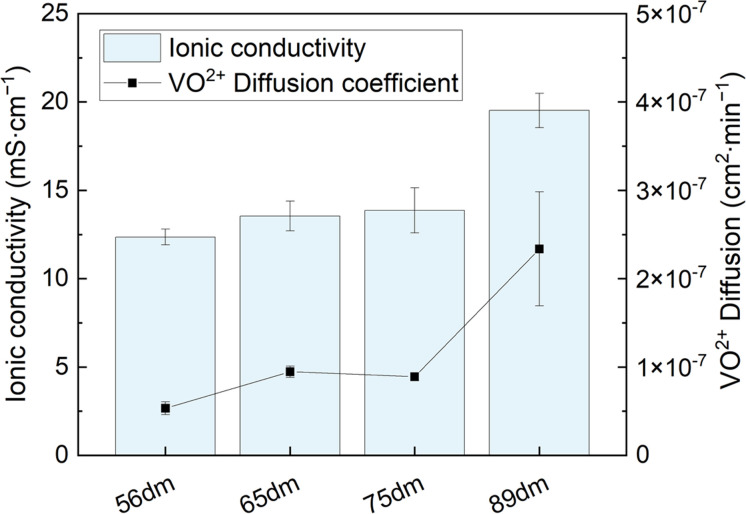
Through-plane ionic conductivity in a 1.6 M vanadium electrolyte (SoC −50%) and VO^2+^ diffusion coefficient of the different HMT-PMBI variants.

Upon contact with the acidic electrolyte, all HMT-PMBI membranes swell around 30% in thickness, with a slight trend seen with higher swelling rates for membranes with a lower degree of methylation, Fig. 3A. This range of thickness swelling is comparable to the swelling of 31% of the singular HMT-PMBI membrane as measured by Shanahan *et al.*^37^ Alongside this swelling degree, the composition of the swollen HMT-PMBI depends on the degree of methylation. Polymers with a lower degree of methylation show a slightly increased uptake of “free acid” inside the film, which describes the acid content in the swollen membrane that is not associated to the backbone of the polymer, such as the HSO_4_^−^ counter-ion. The increase in free acid upon reducing the degree of methylation of the membrane can be explained by the higher concentration of non-methylated benzimidazole moieties with an available lone pair on the nitrogen atom for protonation by the acidic electrolyte. Whereas the fraction of available protonation sites is higher at a low degree of methylation, the opposite is true for the fixed charges on the backbone of polymer. These fixed charges promote the incorporation of water into the membrane through the formation of a hydration shell around the charge.^32^ This trend is also observed upon analyzing the composition of the membrane, with a water content of 34 ± 4 wt% for 56dm, increasing up to 51 ± 1 wt% for 89dm samples. Interestingly, the water content does not appear to change significantly between 65dm and 75dm, with both having a water uptake of ∼41 wt%. In addition to the free H_2_SO_4_, the counter-ion can also be considered as acid bound to the membrane. Taking this into consideration (Fig. 3B), while assuming a complete exchange based on the degree of methylation obtained by ^1^H NMR (Fig. S1–S4†), the total acid content inside the polymer electrolyte can be quantified in terms of molecules of acid per repeating unit. Doing so, a small increase in the total acid content can be observed upon increasing the degree of methylation, which can be attributed to the high amounts of HSO_4_^−^ bound to the methylated benzimidazole core of the polymer. The bound HSO_4_^−^ increases the theoretical ion-exchange capacity of the pristine polymer from 0.4 to 2.1 mmol g^−1^ for respectively 56dm and 89dm, Table S4.† This increase in the total acid content, in combination with the higher hydration of the membrane, plays an important role in the ionic conductivity of the polymer electrolyte. In the case of a vanadium redox flow battery with a sulfuric acid supporting electrolyte, Dai *et al.* showed that the majority of this charge is carried by the H^+^ species when using a polybenzimidazole membrane,^40^ highlighting the importance of both the membrane polymer and the electrolyte on the conductivity. With this in mind, the HMT-PMBI membranes were analyzed for their ionic conductivity in a 1.6 M vanadium electrolyte to mimic the operating conditions in a VRFB cell, Fig. 4.

Similar to the total acid content observed in Fig. 3B, the ionic conductivity shows an increasing trend with the degree of methylation. However, this increase in conductivity is only minor below a 75% degree of methylation, indicating the presence of a baseline-like conductivity that can be attributed to the presence of the vanadium electrolyte imbibed into the membrane. In contrast, a large increase in conductivity is seen upon increasing the degree of methylation to 89%. A similar observation has been reported for fully hydrated HMT-PMBI membranes in water, with the membranes only showing limited conductivity below 75%, and a steep increase upon exceeding 75%.^32^ This increase can be attributed to the presence of a percolating network of aqueous domains associated with the fixed ionic charges in the backbone of the polymer upon exceeding a 75% degree of methylation. Despite a different supporting electrolyte, the general trend of the ionic conductivity in a VRFB remains similar, indicating that the contribution of the HSO_4_^−^ counter-ion on the ionic conductivity of the membrane increases with increasing degree of methylation, thereby showcasing the importance of a high degree of methylation.

Although a high degree of methylation improves the conductivity of the membrane, it can also lead to adverse effects in terms of capacity fading, related to the increase in water uptake as shown in Fig. 3A. To analyze this, the membranes were subjected to VO^2+^ diffusion experiments as described in Section 2.6. For each sample, four experiments were conducted (Fig. S7, Table S6†), with the averaged results shown in Fig. 4. As the degree of methylation of the HMT-PMBI membranes increases, the VO^2+^ diffusion increases alongside with it. This increase closely follows the earlier observed trends for both the water content and ionic conductivity of the samples, with a slight increase from 56dm to 65dm and 75dm followed by a larger jump in crossover for a degree of methylation of 89%. The upward trend in permeability displays that although an improvement in conductivity can be obtained from a higher degree of methylation, this will be accompanied by a worse capacity retention of the flow battery.

In addition to the difference in ionic conductivity and VO^2+^ crossover, the change of the molecular structure and thereby the dissimilar membrane composition, will lead to a change in the structural properties of HMT-PMBI. Wright *et al.* showed that the mechanical properties of HMT-PMBI is highly dependent on the counter-ion and its water content.^33^ Hence, the mechanical properties of HMT-PMBI membranes were screened after equilibration in a 1.6 M vanadium electrolyte (average oxidation state 3.5) to realistically determine its mechanical strength during operation, Table 2.

**Table 2:** Mechanical properties of HMT-PMBI after being equilibrated in 1.6 M vanadium electrolyte (average oxidation state 3.5)

Sample	Tensile stress (MPa)	Elongation at break (%)	Young's modulus (MPa)
56dm	32 ± 3	48 ± 8	345 ± 13
65dm	30 ± 1	53 ± 6	301 ± 30
75dm	28 ± 1	46 ± 6	306 ± 36
89dm	25 ± 1	52 ± 10	259 ± 24

**Fig. 5:**
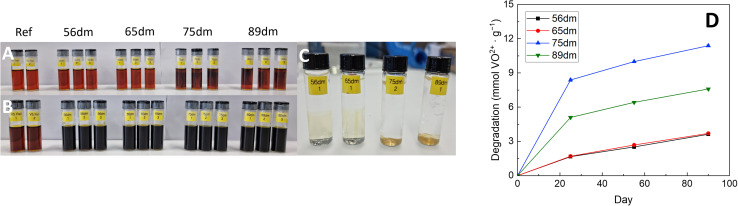
Chemical stability analysis of HMT-PMBI in the 1.6 M VO_2_^+^ electrolyte with the immersed samples at the start of the test (A), at the end of the test after 90 days (B), post-test immersed in 3 M NaCl (C) as well as the degradation of the HMT-PMBI derivatives described in mmol VO^2+^ formed per gram of polymer over these 90 days (D).

Upon increasing the degree of methylation, the membranes become softer, with a decrease seen in the Young's modulus from 345 ± 13 MPa for 56dm to 259 ± 24 MPa for 89dm. Furthermore, this decrease comes with a decrease in the tensile strength of the film from 32 ± 3 MPa to 25 ± 1 MPa, for respectively 56dm and 89dm. This reduction in both the maximum stress and corresponding Young's modulus can be attributed to the higher degree of methylation. Here, the methyl moieties on the backbone act as a spacer between the polymer chains, increasing the free volume inside the polymer by limiting the ordering of the polymer chains, as observed by Jang *et al.* with the addition of alkyl spacers onto an ABPBI backbone.^28^ In addition to the mechanical properties of the membrane, its chemical stability is of great importance for its longevity during operation. In a VRFB, the membranes are subjected to vanadium species in four different oxidation states. Among these, VO_2_^+^ is the most detrimental towards the membrane due to its oxidative strength. Thus, the chemical stability of the HMT-PMBI variants was examined in a fully oxidized 1.6 M VO_2_^+^ electrolyte solution as described in Section 2.8. The immersed samples at the start, end and after as well as the measured degradation during the stability test can be seen in Fig. 5.

Following immersion, HMT-PMBI samples with a degree of methylation of 75% and 89% showed an immediate interaction with the VO_2_^+^ electrolyte, with a discoloration of the electrolyte seen in close proximity to membrane. This discoloration was not witnessed for samples with a lower degree of methylation following immersion, yet only appeared after some days (Fig. S8†), indicating a less severe reaction with the VO_2_^+^ species. After 90 days, all samples show clear signs of VO^2+^ formation as can be seen by the dark color of the electrolyte. This degradation of HMT-PMBI was quantified by analyzing the formation of VO^2+^ by UV-Vis spectroscopy, with samples taken after 25, 55 and 90 days, Fig. 5.

The degradation of all samples show a similar trend, with significant degradation in the first phase of the test, followed by a more linear relationship between day 25 and day 90. Furthermore, the severe interaction of 75dm and 89dm with the VO_2_^+^ electrolyte as witnessed by eye is confirmed by UV-Vis, with both samples having a significantly higher degradation rate than the less methylated membranes. Interestingly, the highest degradation was observed for 75dm, with a decrease seen for 89dm. So far, the exact cause for this higher degradation rate remains uncertain with a more in-depth study being planned. Nevertheless, both 75dm and 89dm experience significantly more degradation than the less methylated 56dm and 65dm. As a consequence, the mechanical integrity of the samples post-test (Fig. 5C) is substantially different, with the 56dm and 65dm membranes remaining intact in the washing step, while the 75dm and 89dm samples broke upon disturbing the solution they were immersed in. In addition, FTIR analysis on the oxidized 56dm and 65dm films showed the presence of two new peaks at 1700 cm^−1^ and 1830 cm^−1^ that cannot be assigned to the acidic electrolyte, Fig. S10 and S11,† indicating the formation of carbonyl moieties and the chemical degradation of the HMT-PMBI polymer. The weakening of both 75dm and to a lesser extend 89dm is also observed in the electrochemical cell, with 75dm samples exhibiting mechanical failure during cycling. Therefore, 75dm membranes had to be excluded from detailed in-cell characterization. The remaining HMT-PMBI variants were analyzed through electrochemical impedance spectroscopy, polarization curves and cycling at varying current densities as described in Section 2.9.

The area specific resistance (ASR) of the HMT-PMBI derivatives in the vanadium flow battery, Table 3, shows the same trend as the *ex situ* measured ionic conductivity, Fig. 4, with an increase in conductivity and therefore a decrease in ASR seen upon increasing the degree of methylation.

**Table 3:** The dry thickness, area specific resistance at −50% SoC and electrolyte crossover towards the negative electrode of the HMT-PMBI variants during the cycling at varying current densities, with Nafion™ NR212 as a commercial reference. All area specific resistance values were corrected for the internal cell resistance of 0.24 ± 0.04 Ω cm^2^

Membrane	Dry thickness (μm)	ASR at −50% SoC (Ω cm^2^)	Crossover towards the negative electrode (mL)
56dm	25	0.25 ± 0.04	4.6
65dm	26	0.22 ± 0.04	5.3
89dm	27	0.17 ± 0.04	15.8
89dm	50	0.27 ± 0.04	8.1
NR212	51	0.22 ± 0.04	−3.4

**Fig. 6:**
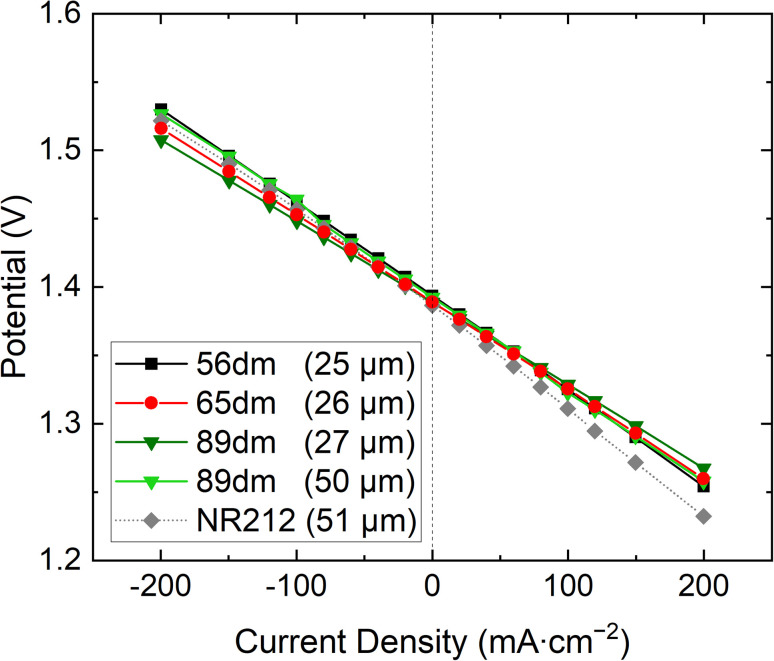
Polarization curves of the various HMT-PMBI membranes at 50% state of charge, with Nafion™ NR212 as commercial reference.

**Fig. 7:**
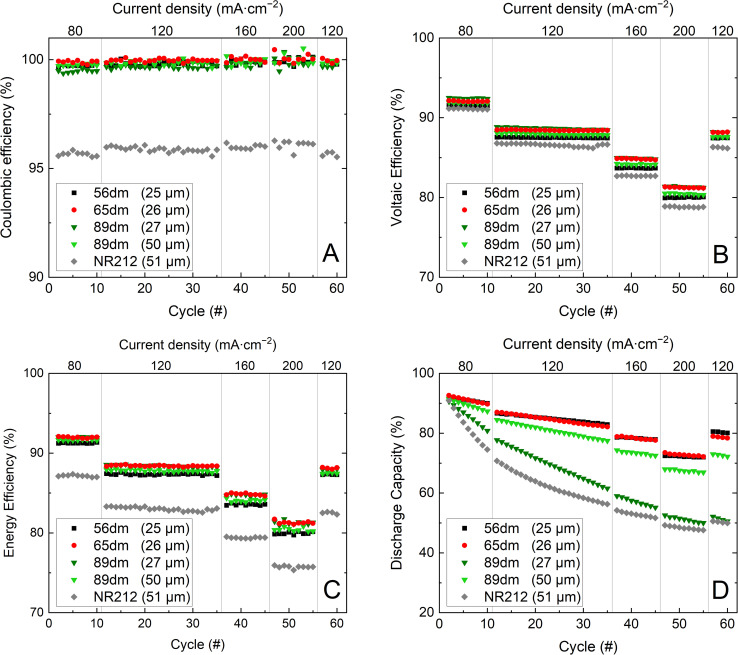
Cycling performance of Nafion™ NR212 and the HMT-PMBI membranes with coulombic efficiency (A), voltaic efficiency (B), energy efficiency (C) and the normalized discharge capacity (D).

Furthermore, the same trend is also seen in the polarization curves, Fig. 6 and S13–S17,† with a lower overpotential obtained for the more methylated materials. Additionally, all HMT-PMBI membranes show a less steep curve compared to Nafion™ NR212, showcasing their excellent performance in a VRFB. When quantifying the performance of the membranes during cycling, Fig. 7, a similar observation can be made, with Nafion™ NR212 having a lower coulombic efficiency, voltaic efficiency and energy efficiency compared to all the HMT-PMBI membranes, while also exhibiting a worse capacity retention.

Comparing the in-cell performance of the HMT-PMBI membranes, all membranes offer a comparable coulombic efficiency, with only a small reduction in coulombic efficiency seen upon increasing the degree of methylation. This decrease in coulombic efficiency can be attributed to the increase in vanadium crossover,^3^ resulting in a more prevalent self-discharge. A larger performance difference is observed in the voltaic efficiency, with the thin 89dm exhibiting the highest voltaic efficiency at the start, albeit dropping faster than that of 65dm due to a combination of the higher chemical degradation and the larger electrolyte crossover. Consequently, the energy efficiency of the 27 μm 89dm is only marginally higher at 80 mA cm^−2^ than that of 65dm while both samples exhibit a similar energy efficiency at the end of the test. The largest difference between samples is seen in the discharge capacity (Fig. 7D). The higher degree of methylation of 89dm results in notably higher capacity fading, whereas the drop in capacity from 56dm to 65dm is less severe. Although the thickness of 89dm can be increased to 50 μm due to its higher ionic conductivity (Fig. 4), the capacity retention remains worse than that of the samples with a lower degree of methylation, while also losing its benefits in the lower ASR and higher voltaic efficiency. This shows that a high degree of methylation is less desirable for VRFB applications. This is notably different from the behavior of HMT-PMBI membranes in other applications such as AEMWE or AEMFC, where a higher degree of methylation is needed to obtain a practical conductivity, while the less methylated samples are not suitable for these applications.^32,33^ Furthermore, as all HMT-PMBI membranes still possess a high energy efficiency at 200 mA cm^−2^, a further increase in current density is possible, with an average drop in energy efficiency of 3.6% seen for 65dm and 89dm and 3.8% for 56dm upon increasing the current density by 40 mA cm^−2^, Fig. S18.† Extrapolating the energy efficiency to higher current densities, an energy efficiency of ∼72% and ∼63% can be expected for 65dm and the thin 89dm at respectively 300 and 400 mA cm^−2^.

**Fig. 8:**
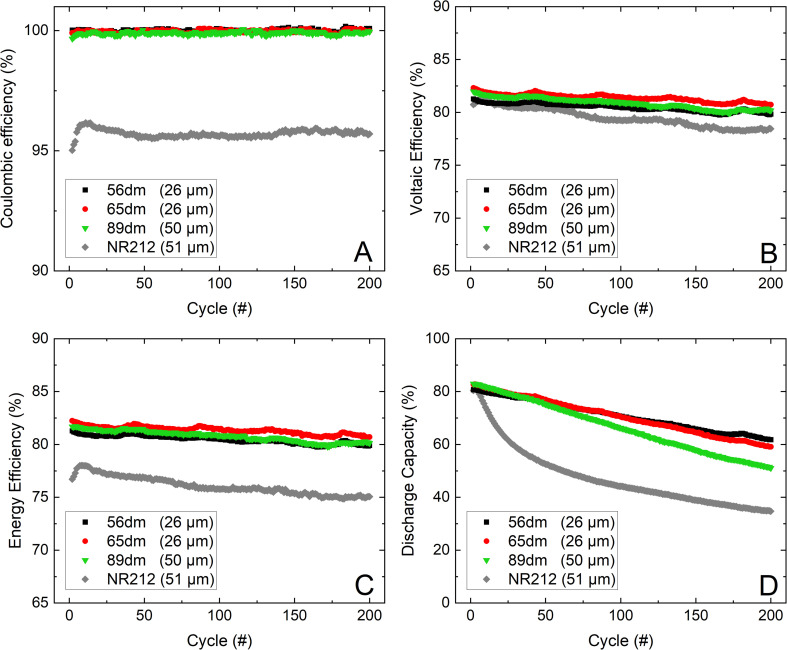
The cycling performance of the HMT-PMBI variants and Nafion™ NR212 over 200 cycles at 200 mA cm^−2^, with the coulombic efficiency (A), voltaic efficiency (B), energy efficiency (C) and normalized discharge capacity (D).

In addition to the characterization of the HMT-PMBI membranes at varying current densities, the more promising samples, respectively 56dm, 65dm and the thicker 89dm, were further analyzed in an extended cycling test at 200 mA cm^−2^, Fig. 8. The highest voltaic efficiency and corresponding energy efficiency in the first phase of the VRFB cycling experiment is obtained by the 26 μm 65dm at ∼82%, slightly higher than the energy efficiency of the 50 μm 89dm and 26 μm 56dm at respectively ∼81.5% and ∼81%. Furthermore, the voltaic efficiency of the 89dm sample drops more rapidly than that of the less methylated samples, which again can be attributed to the higher chemical degradation as observed in Fig. 5 and the more severe drop in capacity (Fig. 8, Table 4), leading to a larger difference in average potential during charging and discharging. As a result, the energy efficiency after 200 cycles at 200 mA cm^−2^ of the 89dm sample is similar to that of the 56dm sample at ∼80%, whereas the 65dm remains the best performing membrane with an energy efficiency of ∼81%. Additionally, the capacity retention follows the same trend as observed during the VRFB cycling test at varying current densities (Fig. 7), with a slight increase in capacity fading from 56dm to 65dm followed by a larger drop in capacity for 89dm, respectively 23% *versus* 28% and 38% capacity loss, Table 4. The higher capacity fading of 89dm in addition to the comparable energy efficiency highlights that HMT-PMBI membranes with a lower degree of methylation are more suited for VRFB applications due to the incorporation of the acidic electrolyte into the membrane. Nevertheless, the performance of all HMT-PMBI variants exceeds that of Nafion™ NR212, with on average a 5% higher energy efficiency over these 200 cycles at 200 mA cm^−2^. Furthermore, while Nafion™ NR212 possesses the lowest capacity retention, it has the least electrolyte imbalance post cycling (Tables 3 and 4), indicating a large discrepancy between the vanadium crossover and the total electrolyte crossover. This dissimilarity results in a different vanadium concentration in the negative and positive electrolytes as shown by Luo *et al.*^41^ This makes the use of a capillary, which connects the electrolyte tanks and allows for continuous rebalancing and therefore a constant volume in each tank, less effective. In contrast, the HMT-PMBI membranes show a much better agreement between the loss of capacity and the electrolyte imbalance, thereby enabling the use of a capillary between the electrolyte tanks to rebalance the flow battery.^42^

**Table 4:** The dry thickness, area specific resistance at −50% SoC, electrolyte crossover and capacity loss of the HMT-PMBI variants and commercial Nafion™ NR212 after 200 cycles at 200 mA cm^−2^. The area specific resistance values were corrected for the internal cell resistance of 0.24 ± 0.04 Ω cm^2^

Membrane	Dry thickness (μm)	ASR at −50% SoC (Ω cm^2^)	Crossover towards the negative electrode (mL)	Crossover (%)	Capacity loss (%)
56dm	26	0.21 ± 0.04	6.8	17	23
65dm	26	0.19 ± 0.04	8.3	21	28
89dm	50	0.23 ± 0.04	10.6	27	38
NR212	51	0.20 ± 0.04	−5.8	14	57

## 4 Conclusion

The suitability of hexamethyl-*p*-terphenyl polybenzimidazolium (HMT-PMBI) membranes for use in a vanadium redox flow battery was investigated by varying the degree of methylation of the HMT-PMBI polymer. An increase in the degree of methylation increases the water content of the polymer electrolyte, thereby contributing to the reduced capacity retention of HMT-PMBI membranes with a higher degree of methylation. In contrast, the acid content increases, improving the ionic conductivity of the membrane and voltaic efficiency during cycling in a vanadium redox flow battery (VRFB). Furthermore, a higher degree of methylation decreases the mechanical robustness and chemical stability in the vanadium electrolyte, making these polymers less suitable for VRFB applications. Membranes with a lower degree of methylation showed an improved cell performance over 200 cycles at 200 mA cm^−2^. Here, the highest energy efficiency was obtained by a 26 μm 65% methylated membrane at ∼82%. Although this sample had the best energy efficiency, a slight improvement in capacity retention is obtained by reducing the degree of methylation to 56%, respectively 77% *versus* 72% capacity retention over 200 cycles. In addition to being fluorine free, the HMT-PMBI membranes outperformed commercial Nafion™ NR212 by an average of ∼5% in terms of energy efficiency over 200 cycles at 200 mA cm^−2^, while also exhibiting a higher capacity retention than the 43% of NR212. With this in mind, HMT-PMBI membranes with a degree of methylation between 56% and 65% can be considered excellent candidates for highly efficient next-generation vanadium flow batteries, thanks to their high energy efficiency and low capacity fading, thereby assisting with the global energy transition towards renewable energy technologies.

## Author contributions

Conceptualization, JCD, SH, LG; data curation, JCD; formal analysis, JCD; funding acquisition, SH, LG; investigation, JCD, BC; methodology, JCD, SH, LG; project administration, SH, LG; resources, SH, LG; supervision, SH, TJS, LG; validation, JCD, BC; visualization, JCD; writing – original draft, JCD; writing – review & editing, BC, SH, TJS, LG. All authors have read and agreed to the published version of the manuscript.

## Conflicts of interest

There are no conflicts to declare.

## Supplementary Material

TA-012-D3TA07212F-s001
