# Supplementary material for: Design of polybenzimidazolium membranes for use in vanadium redox flow batteries
Source: J Mater Chem A Mater. 2024 Feb 8;12(11):6387–98. doi: 10.1039/d3ta07212f (PMC10929584; doi:10.1039/d3ta07212f)
Supplement: TA-012-D3TA07212F-s001 [file TA-012-D3TA07212F-s001.pdf]

Electronic Supplementary Material (ESI) for Journal of Materials Chemistry A.
This journal is © The Royal Society of Chemistry 2024

Design of polybenzimidazolium membranes for use in vanadium redox flow batteries

J.C. Duburg[a,b], B. Chen[b], S. Holdcroft[b], T.J. Schmidt[a,c], L. Gubler*[a]

[a]*Electrochemistry Laboratory, Paul Scherrer Institut, CH-5232, Villigen PSI, Switzerland*

[b]*Simon Fraser University, Department of Chemistry, V5A 1S6 Burnaby, Canada*

[c]*Institute of Molecular Physical Science, ETH Zurich, CH-8093, Zurich, Switzerland*

*lorenz.gubler@psi.ch

Keywords:

Vanadium redox flow battery, polybenzimidazolium, polybenzimidazole, HMT-PMBI membrane, electrochemistry

Table of contents

1. **Polymer characterization** ... 2
2. **Membrane preparation** ... 4
3. **Counter-ion determination** ... 4
4. **Dimensional swelling and electrolyte uptake** ... 4
5. **Ionic conductivity** ... 5
6. **Vanadium permeability** ... 5
7. **Chemical Stability** ... 8
8. **Polarization curves at different states of charge** ... 11
9. **Cycling efficiency versus current density** ... 13
10. **References** ... 14

1. Polymer characterization

Prior to analysis, the HMT-PMBI variants were characterized by ^{1}H nuclear magnetic resonance (NMR) spectroscopy for their degree of methylation. The degree of methylation was calculated according to equation S1, with *x* being the corresponding integration of the uncharged benzimidazole groups (~3.6 ppm) after integrating and setting the area of the charged benzimidazolium groups (~4 ppm) to 12, as described by Wright et al.[1].

$$dm\% = 50\% \left(\frac{1}{1+\frac{x}{6}}\right) + 50\% \qquad \text{(S1)}$$

The ^{1}H NMR spectra of the HMT-PMBI variants can be seen in Figure S1 to Figure S4.

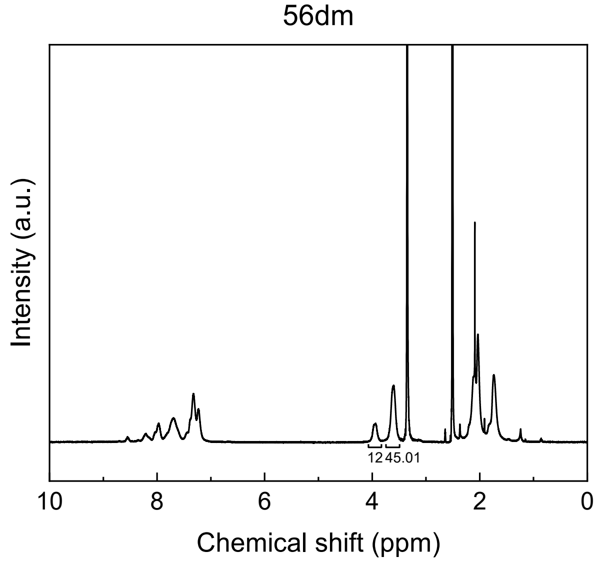

Figure S1. ^{1}H NMR (400 MHz, DMSO-d_6) of HMT-PMBI with a degree of methylation of 56%.

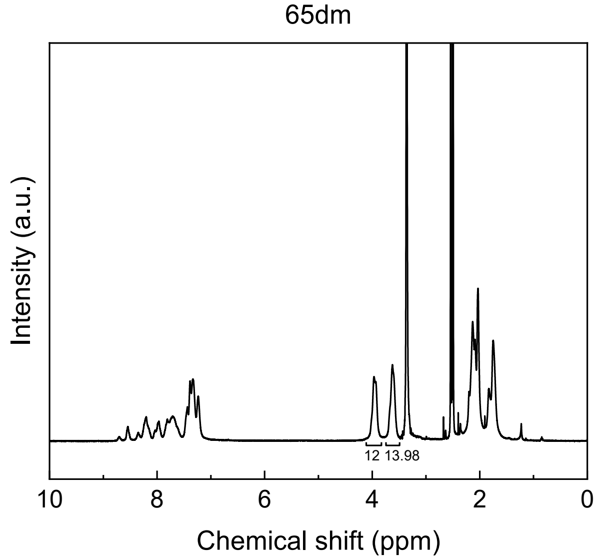

Figure S2. ^{1}H NMR (400 MHz, DMSO-d_6) of HMT-PMBI with a degree of methylation of 65%.

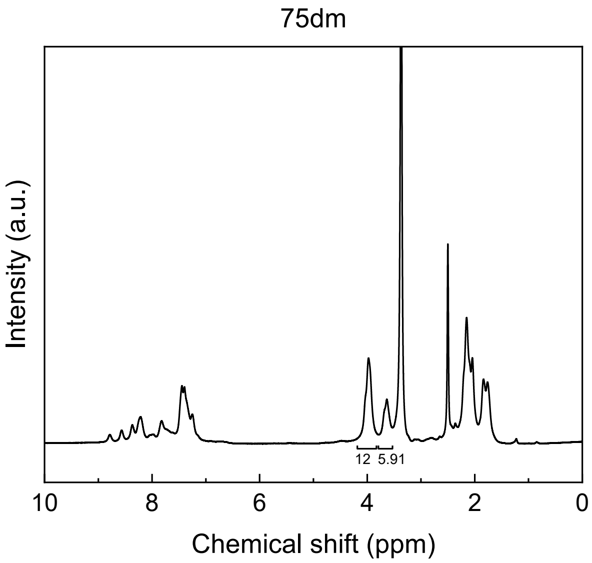

Figure S3. ^{1}H NMR (400 MHz, DMSO-d_6) of HMT-PMBI with a degree of methylation of 75%.

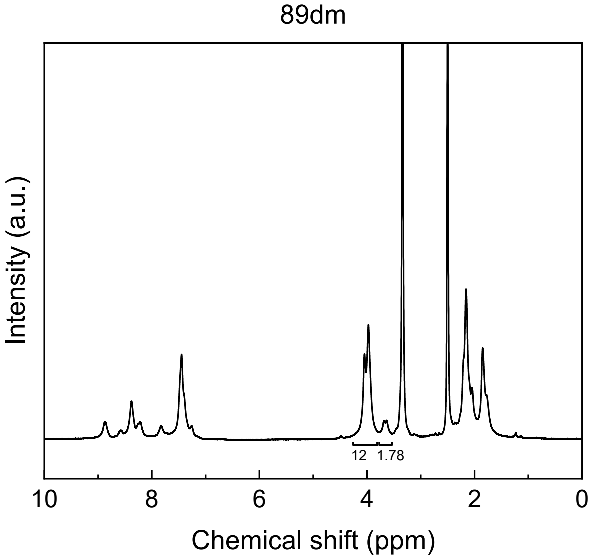

Figure S4. ^{1}H NMR (400 MHz, DMSO-d_6) of HMT-PMBI with a degree of methylation of 89%.

2. Membrane preparation

Membranes were prepared from the HMT-PMBI materials as described in section 2.2 'Membrane preparation' of the main text with the casting conditions for a ~25 µm film given in Table S1.

Table S1. Casting conditions of the HMT-PMBI membranes, with the solvents being dichloromethane (DCM), N-methyl-2-pyrrolidone (NMP) and dimethylsulfoxide (DMSO).

Material	Prefiltration solvent	Weight percentage, casting solvent	Blade gap (µm)	Evaporation temperature	Evaporation time
56dm	DCM	11.0%, NMP	400	80 °C	Overnight
65dm	DCM	12.5%, NMP	400	80 °C	Overnight
75dm	Methanol	11.0%, DMSO	350	80 °C	Overnight
89dm	N.A.	N.A.	N.A.	N.A.	N.A.

3. Counter-ion determination

Table S2. The detection limits of the pristine and treated 89dm XRF analysis.

Component	Pristine 89dm (wt.%)	Detection limit pristine 89dm (wt.%)	Treated 89dm (wt.%)	Detection limit treated 89dm (wt.%)
S	0.2	0.00026	1.4	0.00044
Cl	1.3	0.0013	< 0.1	0.00063
I	0.1	0.00051	< 0.1	0.00048
CH_2	98.3	-	98.5	-

4. Dimensional swelling and electrolyte uptake

The dimensional swelling and electrolyte uptake of the HMT-PMBI membranes with a varying degree of methylation was measured as described in section 2.4 'Dimensional swelling and electrolyte uptake' of the main text. The electrolyte uptake in terms of weight content and dimensional swelling can be seen in Table S3, with the electrolyte uptake in terms of mols per mol polymer reported in Table S4.

Table S3. Water uptake, free acid uptake, thickness swelling and in-plane swelling of HMT-PMBI membranes with various degrees of methylation upon immersion in 2 M H_2SO_4, with the uptake reported in terms of weight content.

Material	Free H_2O uptake (wt. %)	Free H_2SO_4 uptake (wt. %)	Thickness swelling (%)	In-plane swelling (%)
56dm	34.0 ± 4.0	35.4 ± 0.8	33.7 ± 0.7	3.2 ± 0.2
65dm	40.7 ± 1.6	28.7 ± 0.7	34.4 ± 1.3	6.3 ± 0.6
75dm	41.6 ± 1.8	21.0 ± 0.5	29.5 ± 2.2	7.3 ± 1.9
89dm	51.0 ± 0.8	13.4 ± 0.2	29.8 ± 0.6	10.6 ± 0.9

Table S4. Water uptake, free acid uptake, bound acid uptake and ion-exchange capacity of HMT-PMBI membranes with various degrees of methylation upon immersion in 2 M H_2SO_4 in terms of mols per mol polymer.

Material	Free H_2O (mol · mol^{-1} polymer)	Free H_2SO_4 (mol · mol^{-1} polymer)	Bound HSO_4^- (mol · mol^{-1} polymer)	IEC (mmol HSO_4^-·g^{-1})
56dm	10.74 ± 1.31	2.16 ± 0.05	0.24	0.4
65dm	13.02 ± 0.54	1.87 ± 0.05	0.60	0.9
75dm	13.67 ± 0.62	1.47 ± 0.03	1.01	1.5
89dm	17.74 ± 0.24	1.02 ± 0.01	1.54	2.1

5. Ionic conductivity

The through-plane ionic conductivity of the HMT-PMBI membranes was measured as described in section 2.5 of the main text, with the results depicted in Table S5.

Table S5. Through-plane ionic conductivity of the different HMT-PMBI variants in 2 M H_2SO_4 and a 1.6 M vanadium electrolyte (oxidation state 3.5).

Material	Ionic conductivity in 2 M H_2SO_4 (mS·cm^{-1})	Ionic conductivity in vanadium electrolyte (mS·cm^{-1})
56dm	27.7 ± 4.2	12.4 ± 0.4
65dm	30.9 ± 3.2	13.5 ± 0.8
75dm	39.7 ± 5.8	13.9 ± 1.3
89dm	54.8 ± 10.7	19.5 ± 1.0

6. Vanadium permeability

As described in section 2.6 'VO^{2+} diffusion', the VO^{2+} concentration was determined using a VO^{2+} calibration curve (Table S5). This calibration curve was prepared by diluting a 1000 mg vanadium standard ($VOSO_4$ in 8.6% H_2SO_4, Titrisol®) with 2 M H_2SO_4 to a vanadium concentration of 0.01 M, 0.02 M, 0.03 M and 0.04 M. Subsequently, the calibration curve was prepared by analyzing the UV-Vis absorbance of the solutions at 765 nm and plotting these versus their concentration.

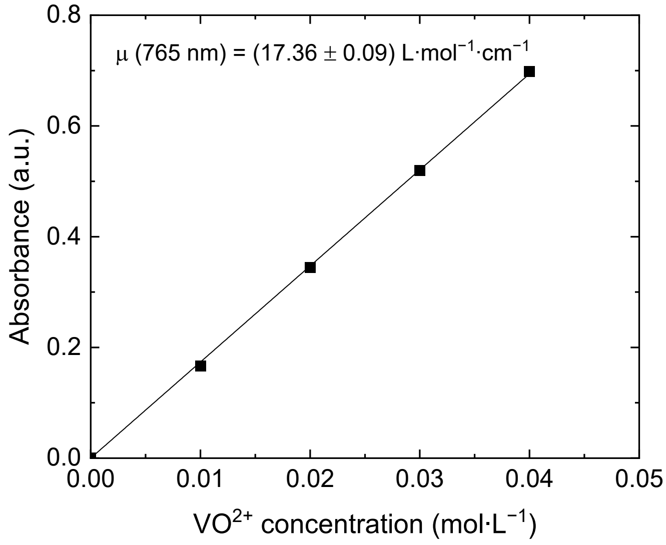

Figure S5. $VOSO_4$ calibration curve, measured through the dilution of a 1000 mg V, ($VOSO_4$ in 8.6% H_2SO_4) Titrisol® standard in 2 M H_2SO_4.

The VO^{2+} diffusion experiments were carried out in a home-built diffusion cell, Figure S6, as described in section 2.6 'VO^{2+} diffusion'. Four individual measurements were done for each HMT-PMBI membrane, Figure S7, with each test contributing to the averaged result shown in the main text. The measured diffusion coefficient corresponding to each individual test, including the averaged diffusion coefficient and its corresponding error given as the standard deviation from each individual test can be seen in Table S6.

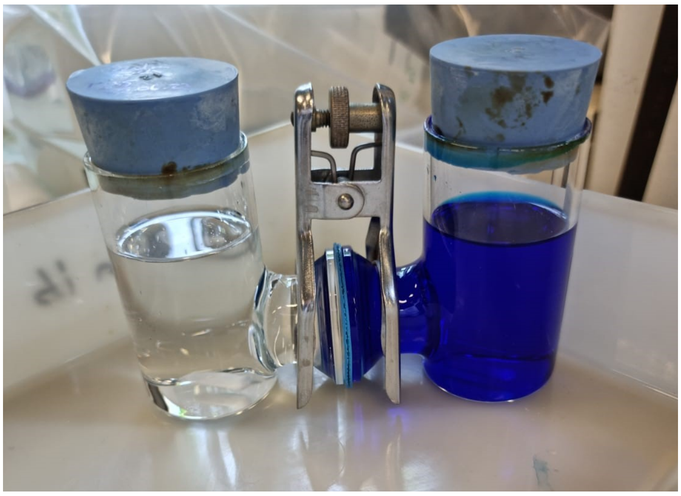

Figure S6. Home-built VO^{2+} diffusion cell.

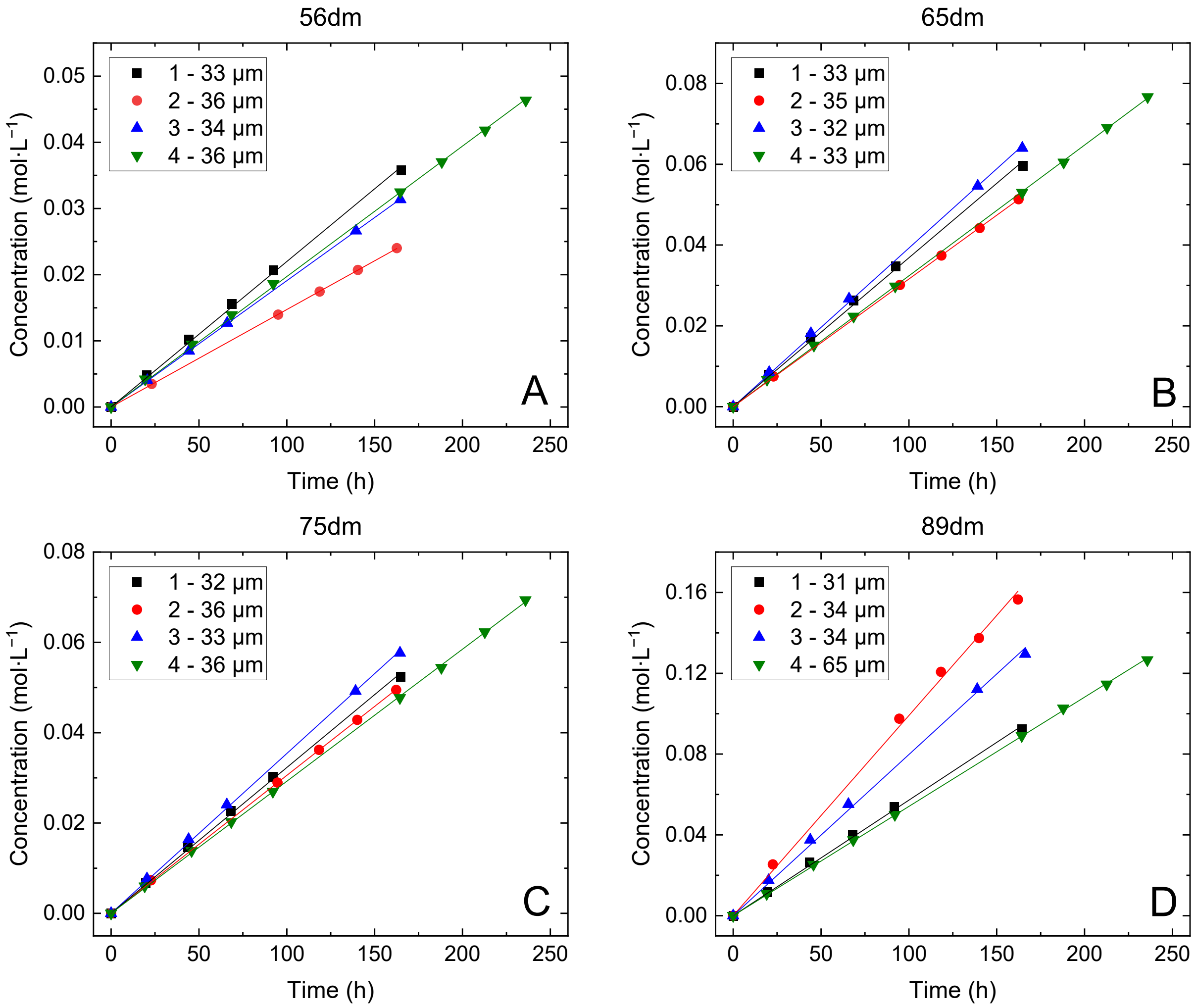

Figure S7. The four individual VO^{2+} diffusion measurements of 56dm (A), 65dm (B), 75dm (C) and 89dm (D) with the given membrane thickness being the swollen thickness at the end of the test.

Table S6. The VO^{2+} diffusion coefficients of each individual measurement and the corresponding averaged diffusion coefficient.

Measurement	56dm $D_{VO^{2+}}$ (10^{-9} $cm^2 \cdot min^{-1}$)	65dm $D_{VO^{2+}}$ (10^{-9} $cm^2 \cdot min^{-1}$)	75dm $D_{VO^{2+}}$ (10^{-9} $cm^2 \cdot min^{-1}$)	89dm $D_{VO^{2+}}$ (10^{-9} $cm^2 \cdot min^{-1}$)
1	60 ± 1	99 ± 2	85 ± 2	147 ± 3
2	43 ± 1	90 ± 1	91 ± 1	278 ± 6
3	53 ± 1	101 ± 3	94 ± 2	223 ± 5
4	57 ± 2	89 ± 6	86 ± <1	287 ± 3
Average	53 ± 7	95 ± 6	89 ± 4	234 ± 64

7. Chemical Stability

The chemical stability of the HMT-PMBI variants was determined as described in section 2.8 of the main text. The samples in solution at the start and during this test after 25, 55 and 90 days can be seen in Figure S8.

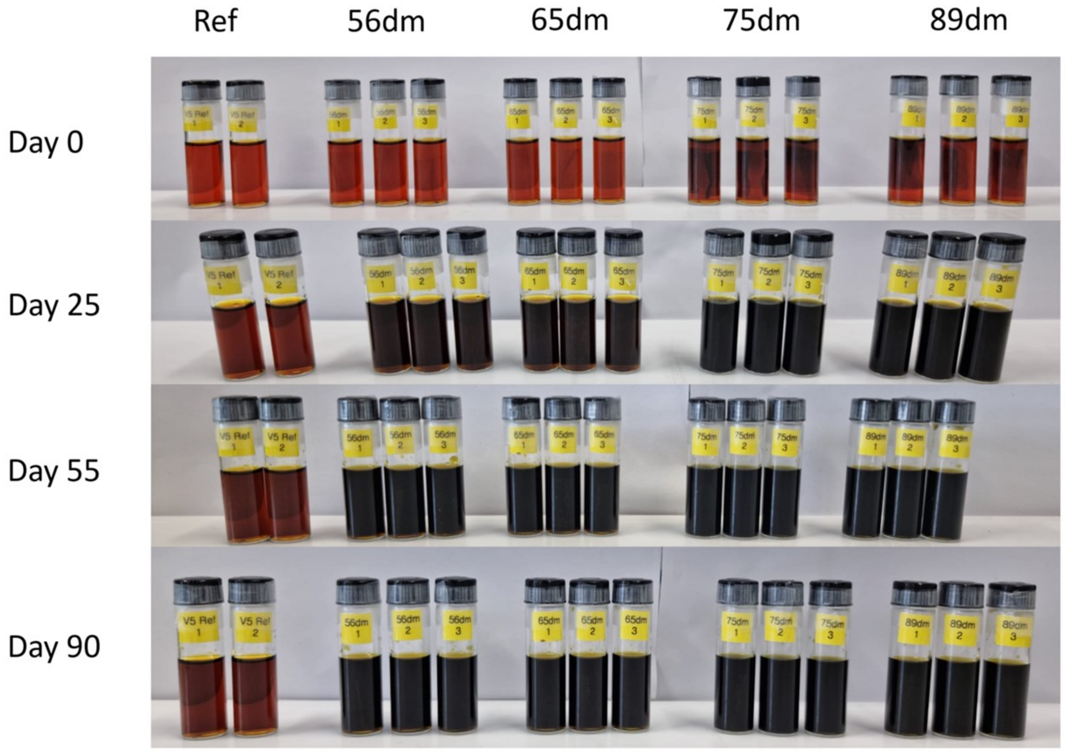

Figure S8. The HMT-PMBI samples during the chemical stability analysis in the 1.6 M VO_2^+ electrolyte, with the immersed samples at the start of the test and after day 25, day 55 and day 90.

In addition to the observed degradation of the HMT-PMBI membranes by VO_2^+ as shown in Figure 5, the membranes were analyzed after the chemical stability test by Fourier-transform infrared (FTIR) spectroscopy and ^{1}H nuclear magnetic resonance (NMR) spectroscopy. FTIR spectra of the oxidized 75dm and 89dm films could not be measured as a result of their mechanical disintegration, with the spectra of the oxidized 56dm and 65dm being depicted in Figure S10 and Figure S11, respectively. Furthermore, the FTIR spectra of the pristine HMT-PMBI films can be seen in Figure S9.

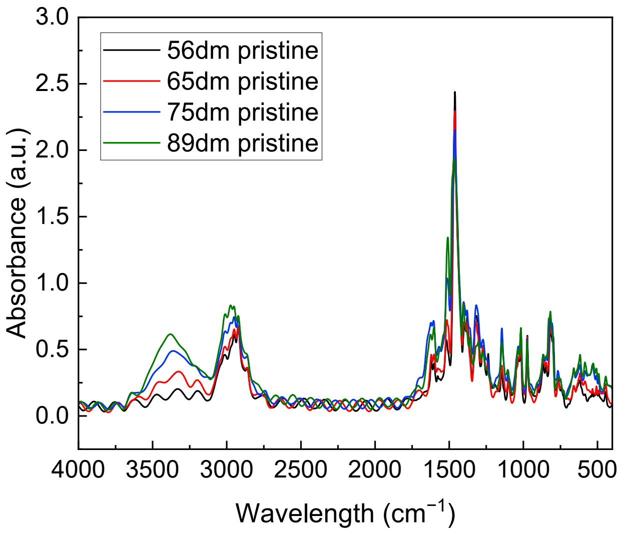

Figure S9. FTIR spectra of the pristine HMT-PMBI samples prior to the chemical stability test.

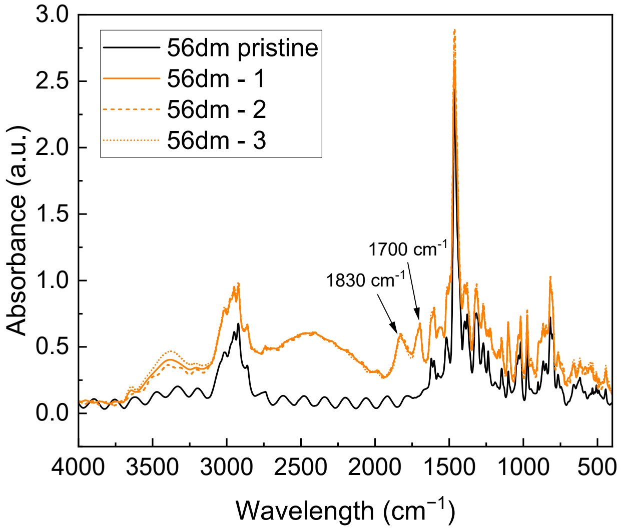

Figure S10. FTIR spectra of the pristine and three oxidized 56dm films.

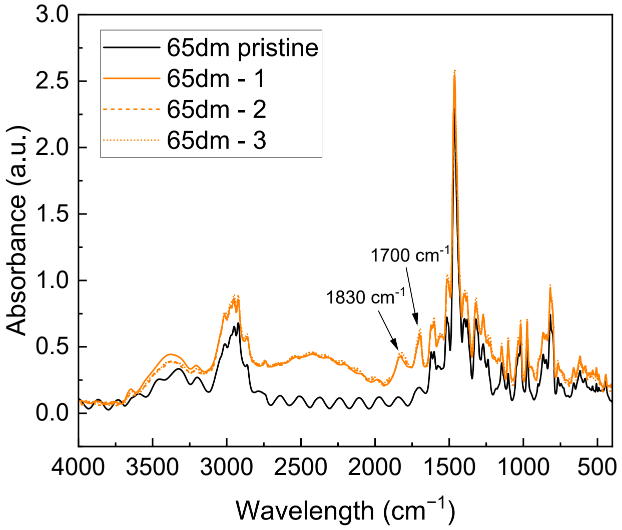

Figure S11. FTIR spectra of the pristine and three oxidized 65dm films.

The FTIR spectra of the oxidated 56dm and 65dm both show the presence of two new peaks at 1700 cm^{-1} and 1830 cm^{-1} that cannot be assigned to the acidic electrolyte, indicating the formation of carbonyl moieties and the chemical degradation of the HMT-PMBI polymer. The signal appearing between 2000 – 2700 cm^{-1} can be assigned to the N^+-H group formed by the protonation of the benzimidazole backbone and is therefore not an indication of polymer degradation[2].

Despite the new FTIR bands observed in the oxidized samples, only a slight peak shift is observed in the ^{1}H NMR spectra of all oxidized HMT-PMBI polymers, Figure S12, with no newly formed distinct peaks. The lack of new signals is hypothesized to be the result of the water solubility of most degradation products, therefore leaving only the less degraded polymer after the washing step.

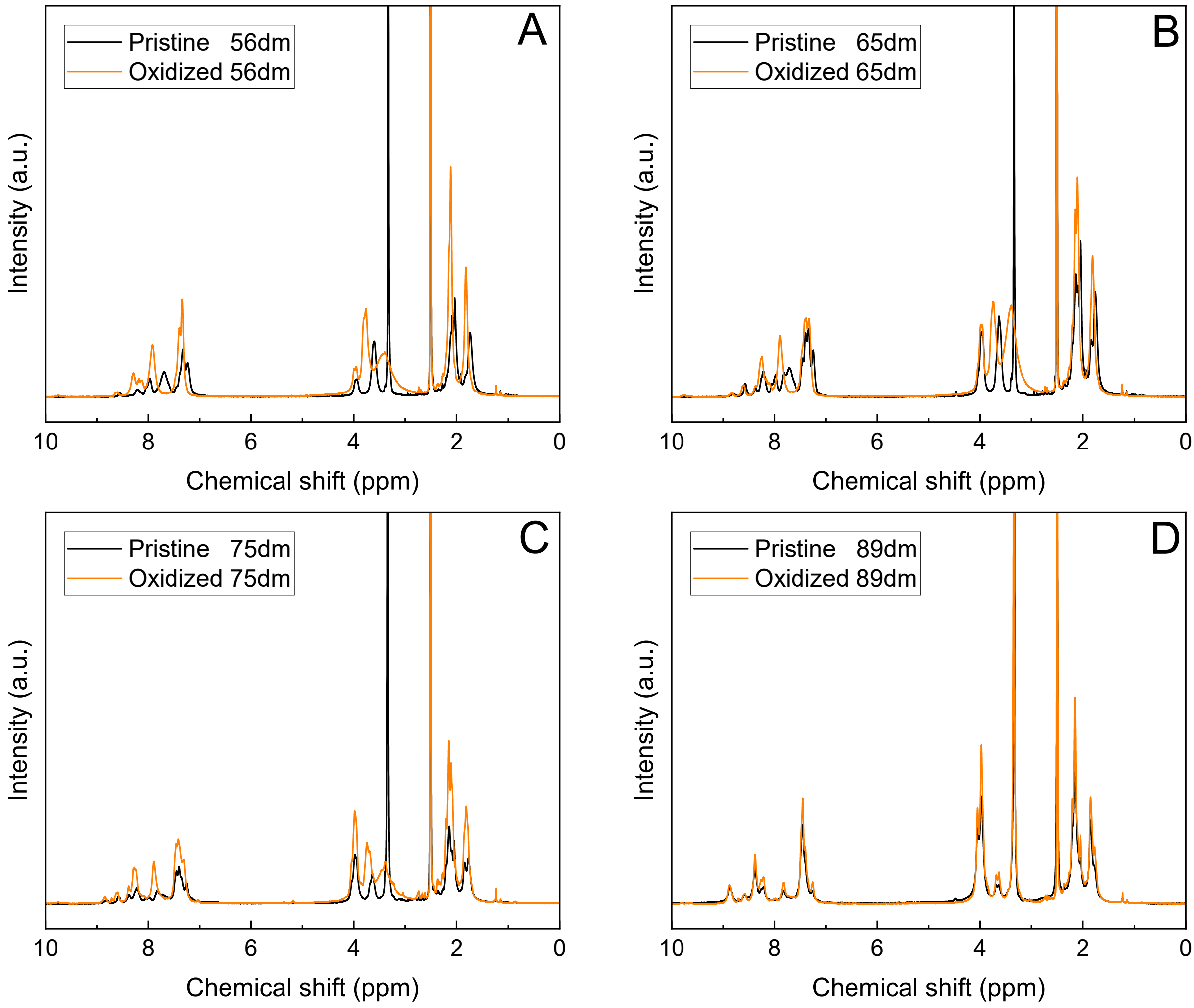

Figure S12. ^{1}H NMR spectra (400 MHz, DMSO-d_6) of the HMT-PMBI samples before and after the chemical stability test

8. Polarization curves at different states of charge

The polarization curves of Nafion™ NR212 and the HMT-PMBI were recorded as described in section 2.9 'VRFB cell cycling' of the main text. The polarization of each membrane at a state of charge (SoC) of 20, 30, 50, 70 and 90% can be seen in Figure S13-S17.

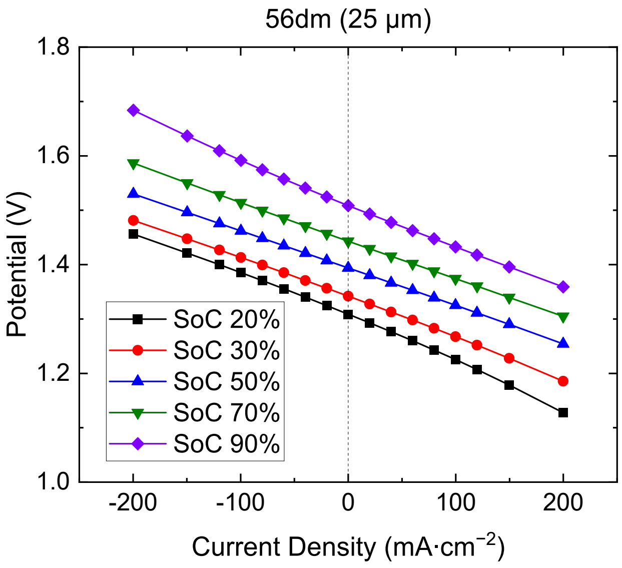

Figure S13. Polarization curves at a state of charge of 20, 30, 50, 70 and 90% of a 56dm membrane with a dry thickness of 25 µm.

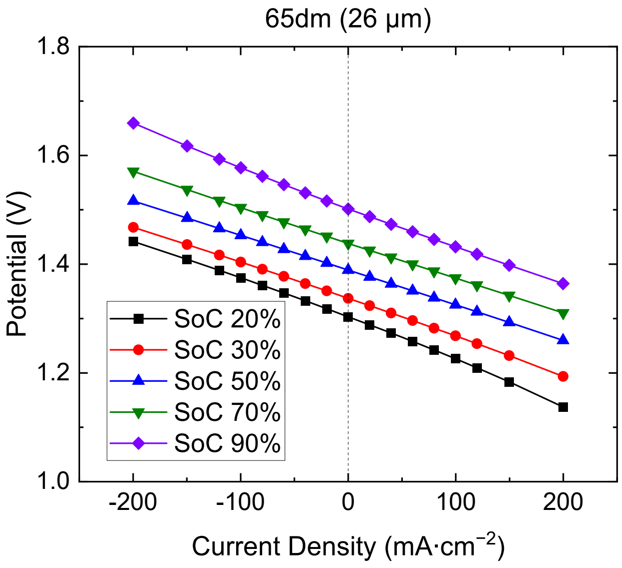

Figure S14. Polarization curves at a state of charge of 20, 30, 50, 70 and 90% of a 65dm membrane with a dry thickness of 26 µm.

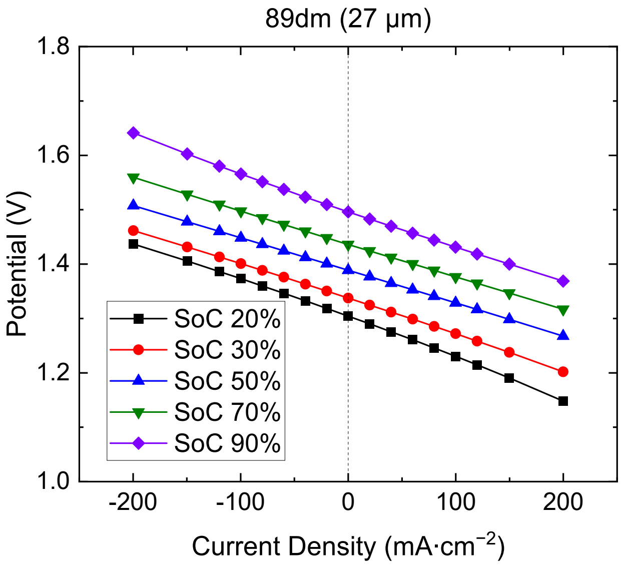

Figure S15. Polarization curves at a state of charge of 20, 30, 50, 70 and 90% of an 89dm membrane with a dry thickness of 27 µm.

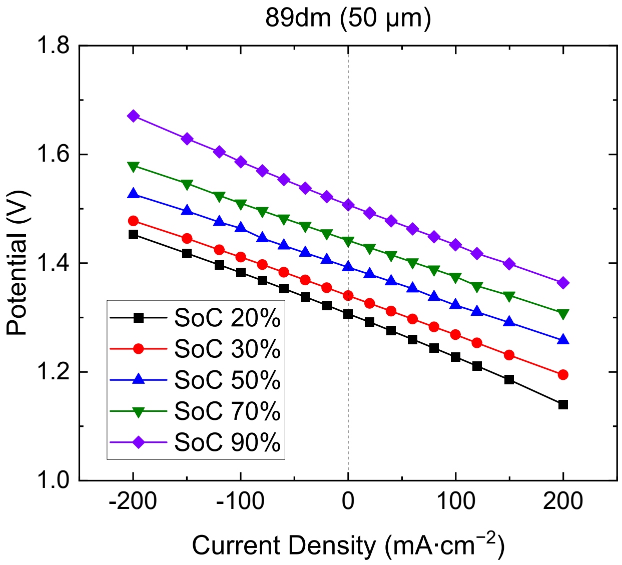

Figure S16. Polarization curves at a state of charge of 20, 30, 50, 70 and 90% of an 89dm membrane with a dry thickness of 50 µm.

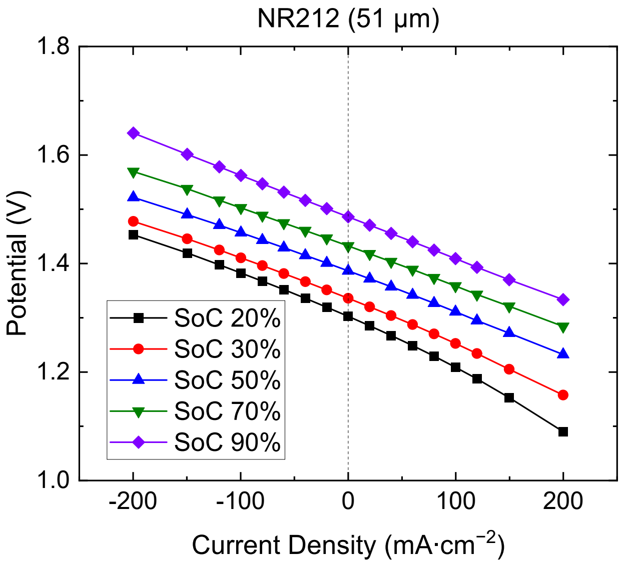

Figure S17. Polarization curves at a state of charge of 20, 30, 50, 70 and 90% of a Nafion™ NR212 membrane with a dry thickness of 51 µm.

9. Cycling efficiency versus current density

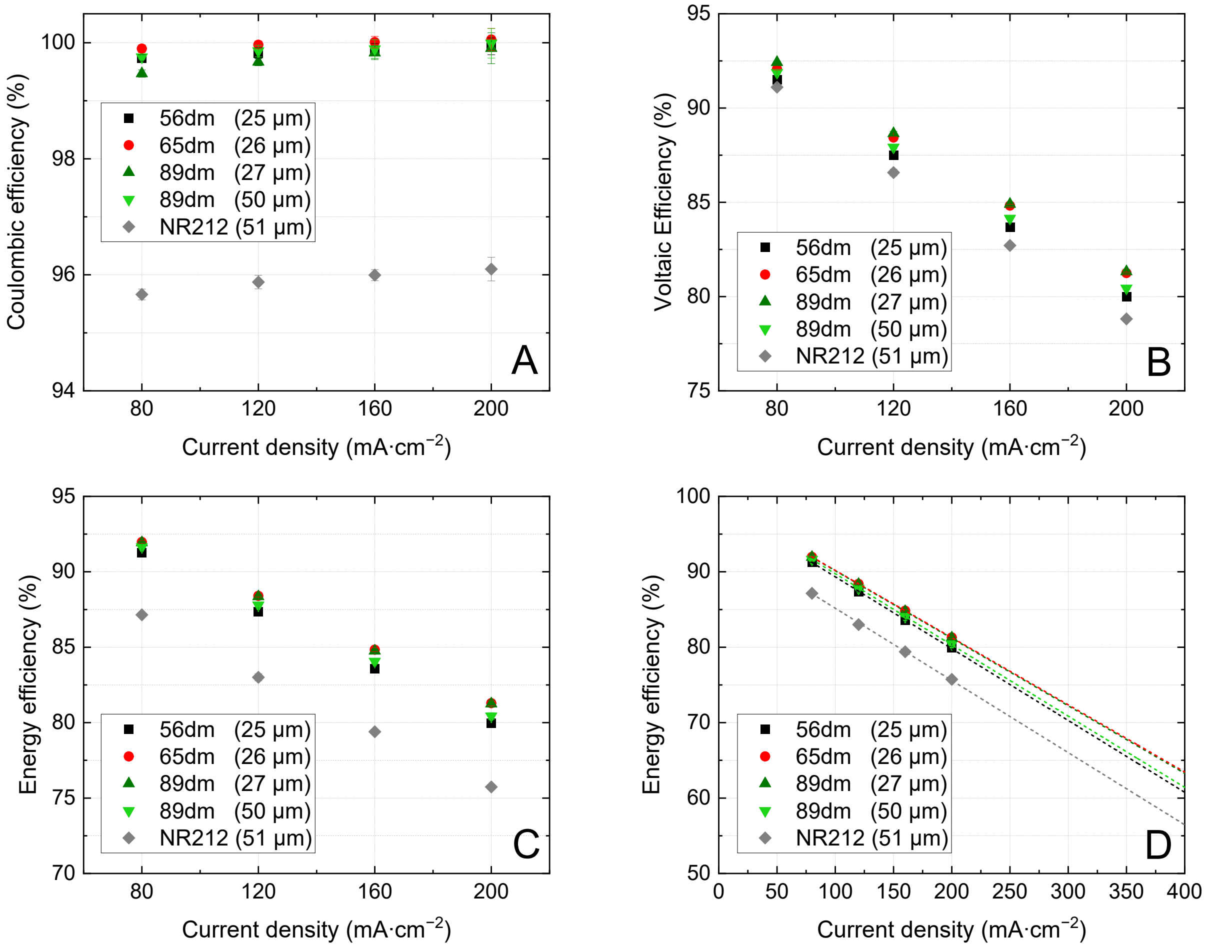

Figure S18. Cycling performance of Nafion™ NR212 and the HMT-PMBI membranes between 80 and 200 mA·cm^{-2} with coulombic efficiency (A), voltaic efficiency (B), energy efficiency (C) and the extrapolated energy efficiency at higher current densities (D).

10. References

1. A. G. Wright and S. Holdcroft, *ACS Macro Letters*, 2014, **3**, 444-447.
2. L. Gubler, D. Vonlanthen, A. Schneider and F. J. Oldenburg, *Journal of The Electrochemical Society*, 2020, **167**, 100502.
